# Measurement Entropy and Conditional Information in Time-Reversed Young Interferometry

**DOI:** 10.3390/e28101093

**Published:** 2026-10-08

**Authors:** Jianming Wen

**Affiliations:** Department of Electrical and Computer Engineering, Binghamton University, Binghamton, NY 13902, USA; jwen7@binghamton.edu

**Keywords:** time-reversed Young interferometry, Shannon entropy, conditional information, Kullback–Leibler divergence, mutual information, optical reciprocity, statistical optics, optical coherence

## Abstract

Time-reversed Young (TRY) interferometry holds a detector channel fixed while a source coordinate is scanned or programmed. We describe this measurement through a single accepted source–detector probability law obtained from scalar Fresnel propagation, the programmed source prior, and detector acceptance. The conventional fixed-source Young distribution, the source and detector marginals, and the fixed-detector TRY distribution are then different reductions of that common record. Standard information measures can therefore be assigned without ambiguity to the probability law actually measured. For a symmetric double-slit response with matched source and detector windows, the fixed-source and fixed-detector conditional distributions have the same entropy; the physically useful information from detector conditioning is instead expressed by grid-stable entropy deficits and Kullback–Leibler information gains. The canonical example gives a modest prior-to-posterior information gain and establishes a transparent baseline for more selective source programming, detector modes, or operating points. Finite slit width, finite-source element width, finite detector pixels, and finite-count entropy estimation are included to connect the ideal model with experiments. A phase scan then shows that conditional entropy and Fisher information characterize different properties of the same likelihood, while a two-mode coherence model separates record entropy from optical coherence entropy and distinguishes coherent superposition from incoherent mixture. The framework gives a unified statistical interpretation of fixed-detector, source-programmed Young interferometry while keeping optical reciprocity, Bayesian conditioning, local parameter sensitivity, and quantum state disturbance conceptually distinct.

## 1. Introduction

Young interference is normally read on the detector side of an optical system. A prescribed source illuminates two paths, the detector coordinate is sampled, and the accumulated counts form a detector-plane distribution [1,2,3,4,5]. This source-to-receiver view also underlies broader modal and channel descriptions of optical propagation [6,7]. Time-reversed Young (TRY) interferometry exchanges the operational roles of the two endpoints: the detector is held at a selected output channel while a source coordinate is scanned or programmed, so the response is reconstructed over a retained source label rather than over detector position [8]. A recent statistical treatment formulates this source-labeled response in terms of photon-counting statistics and fixed-detector likelihoods [9]; related work has developed Bayesian source-prior engineering [10] and differential source-basis estimation [11].

The source-space readout raises a precise entropy question. A detector histogram and a source-label histogram can both be normalized and assigned Shannon entropies, but they are not automatically the same probability distribution written in different coordinates. A conventional fixed-source Young measurement conditions on a source setting and resolves detector outcomes. TRY conditions on a detector outcome and resolves source labels. If several source settings are programmed and their labels are then ignored, the result is a third object: the detector marginal of the source ensemble. These operations must be separated before their entropies are compared. Related links between coherence, estimation, and imaging resolution have been studied in broader optical settings [12,13,14,15,16].

The optical response alone does not assign probabilities to the source labels. A scan becomes a statistical ensemble only after a source-programming measure has been specified, for example, through the relative dwell time or trial frequency assigned to each source setting. This measurement-level description is consistent with information-theoretic treatments of measurement systems [17] and with recent field-level developments in electromagnetic information theory [18]. The entropy considered below belongs to the classical source–detector record formed after source programming, propagation, detector acceptance, and normalization rather than to the optical field.

Shannon entropy, relative entropy, mutual information, and Fisher information describe different statistical properties of a probability law [19,20,21,22,23,24,25]. For continuous coordinates, differential entropy depends on the coordinate scale, whereas thermodynamic entropy refers to a different physical quantity [26,27,28,29]. The optical propagation is therefore first formulated in continuous coordinates and subsequently reduced to discrete source settings and detector channels for the Shannon entropy analysis.

The analysis therefore follows one sequence from optics to information. Scalar Fresnel propagation first defines the source–detector transfer. Source programming and detector acceptance then convert that transfer into a normalized joint probability law. Marginalization or conditioning produces the detector marginal, the accepted source marginal, the fixed-source Young distribution, and the fixed-detector TRY distribution. Entropy, relative information, mutual information, and local sensitivity are introduced only after these probability laws are specified. Detailed derivations, finite-width generalizations, numerical sums, and notation are provided in the Supplementary Materials.

Optical reciprocity and statistical conditioning enter at different levels of the description. Reciprocity constrains the Green function of physically exchanged source–receiver modes [30,31,32], whereas the symmetry of mutual information is a property of any joint probability distribution. Bayesian inversion is likewise prior-dependent [33,34,35]. Here, “time-reversed Young” denotes the source–detector-exchanged measurement geometry introduced in Ref. [8]; it does not imply the microscopic time reversal of a stochastic process. The fixed-detector distribution derived below is a conditional source-label distribution generated by the optical transfer and the programmed source prior.

Throughout the joint-record model, the source label is a classical preparation setting, and the detected data are classical counts. This distinction matters when the language of quantum measurement is invoked: sequential backaction, invasiveness, and tests of realism require a quantum state together with a measurement instrument or state-update map [36,37,38]. The two-mode coherence operator introduced later is used only to compare the optical coherence entropy with the entropy of the classical measurement record; it is not a state-update model for the source-label scan.

These ingredients define a single probability hierarchy. Detector-side and source-side entropies belong to different reductions of the same accepted joint record, conditioning on a selected detector updates the programmed source distribution, and averaging that update separates source–detector correlation from the acceptance-induced reshaping of the source ensemble. This hierarchy gives a direct measurement-level interpretation of the entropy and information quantities used below.

**Figure 1:**
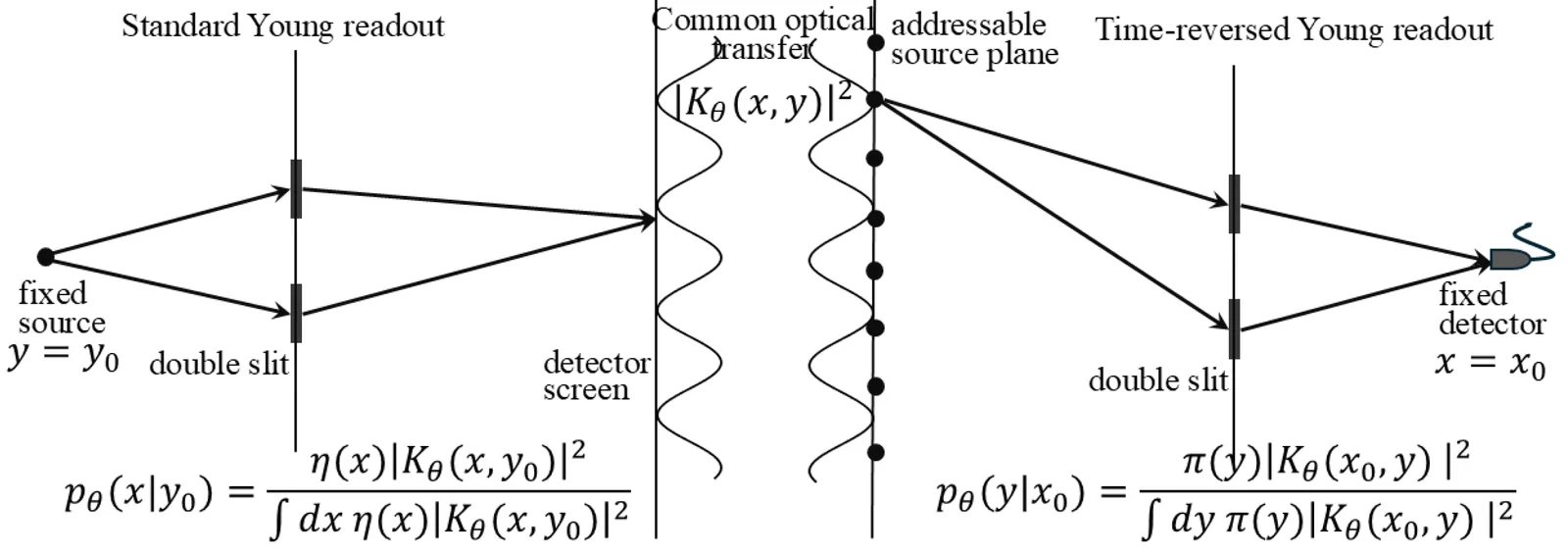
Conceptual comparison of the two conditional readouts generated by the same source–detector optical transfer. Left: a standard Young measurement fixes the source at $y_{0}$ and normalizes the accepted intensity over detector position *x*, yielding $p_{\theta }\left(x|y_{0}\right)$. Right: TRY fixes the detector at $x_{0}$ and normalizes the accepted response over the programmed source coordinate *y*, yielding $p_{\theta }\left(y|x_{0}\right)$. The common optical ingredient is $|K_{\theta }{\left(x,y\right)|}^{2}$; the detector acceptance η(x) and source prior π(y) enter the corresponding conditional laws. Ensemble marginals are obtained separately by integrating or summing the joint record over one coordinate.

Figure 1 summarizes the measurement organization. The same optical transfer connects source and detector coordinates, but the two experiments retain and normalize different variables.

## 2. Optical and Statistical Model

### 2.1. Scalar Propagation and the Double-Slit Response

Let *y* denote the transverse source coordinate, *u* the coordinate in the aperture plane, and *x* the detector coordinate. The source-to-aperture and aperture-to-detector distances are $L_{s}$ and $L_{d}$. For monochromatic scalar light of wavelength λ, the wavenumber is k=2π/λ. If $T_{\theta }\left(u\right)$ is the complex aperture transmission, where θ denotes any physical parameter that changes the optical response, paraxial Fresnel propagation gives [39]

(1) $$K_{\theta }\left(x,y\right)=\int du\;T_{\theta }\left(u\right)\exp\;\left[\frac{ik}{2L_{s}}{\left(u-y\right)}^{2}+\frac{ik}{2L_{d}}{\left(x-u\right)}^{2}\right].$$

A coordinate-independent Fresnel prefactor is omitted because it cancels from every normalized probability below. Equation (1) is the optical starting point in this paper.

For a narrow double slit centered at $u_{\pm }=\pm d/2$, write

(2) $$T_{\theta }\left(u\right)=a_{+}e^{i\phi_{+}\left(\theta \right)}\delta \left(u-d/2\right)+a_{-}e^{i\phi_{-}\left(\theta \right)}\delta \left(u+d/2\right),$$

where *d* is the slit separation, $a_{\pm }\geq 0$ are the amplitude transmissions, and $\phi_{\pm }$ are additional path phases. Substitution of Equation (2) into Equation (1) gives

(3) $$\begin{array}{c} K_{\theta }\left(x,y\right)=C\sum_{s=\pm }a_{s}\exp\;\left[\frac{ik}{2L_{s}}{\left(u_{s}-y\right)}^{2}+\frac{ik}{2L_{d}}{\left(x-u_{s}\right)}^{2}+i\phi_{s}\left(\theta \right)\right], \end{array}$$

where *C* is the common propagation factor omitted in Equation (1).

The phase difference between the two terms in Equation (3) is obtained directly by subtraction:

(4) $$\begin{array}{cc} \Delta \Phi_{\theta }\left(x,y\right) & =\frac{k}{2L_{s}}\;\left[{\left(d/2-y\right)}^{2}-{\left(-d/2-y\right)}^{2}\right]+\frac{k}{2L_{d}}\;\left[{\left(x-d/2\right)}^{2}-{\left(x+d/2\right)}^{2}\right]+\Delta \phi_{\theta }\\ & =-kd\left(\frac{y}{L_{s}}+\frac{x}{L_{d}}\right)+\Delta \phi_{\theta }, \end{array}$$

where $\Delta \phi_{\theta }=\phi_{+}\left(\theta \right)-\phi_{-}\left(\theta \right)$. Consequently,

(5) $$|K_{\theta }{\left(x,y\right)|}^{2}={\left|C\right|}^{2}\left[a_{+}^{2}+a_{-}^{2}+2a_{+}a_{-}\cos\Delta \Phi_{\theta }\left(x,y\right)\right].$$

Equations (4) and (5) make the two-coordinate structure explicit. Holding *y* fixed and varying *x* gives a detector-space Young response. Holding *x* fixed and varying *y* gives the source-space TRY response. No separate fringe law is required.

The delta function slits are the narrow aperture limit of finite transmitting windows. For slit width *w*, the Fresnel integral contains the same relative two-path phase together with a single-slit envelope; in the Fraunhofer limit, that envelope is proportional to ${\mathrm{sinc}}^{2}\left[\left(kw/2\right)\left(y/L_{s}+x/L_{d}\right)\right]$. Finite slit width therefore reduces high-spatial-frequency structure in both detector-space and source-space conditionals. The point-slit approximation is accurate when the envelope varies slowly over the sampled window; the finite-width expression is given in Supplemental Section S2.4.

A programmed source setting can also have finite transverse extent. If source label *j* excites a normalized spatial profile $q_{j}\left(y\right)$ rather than a point at $y_{j}$, an incoherent source element contributes the locally averaged transfer $\int dy\;q_{j}\left(y\right){\left|K_{\theta }\left(x,y\right)\right|}^{2}$. For a coherent source mode with amplitude profile $f_{j}\left(y\right)$, the corresponding amplitude is $\int dy\;f_{j}\left(y\right)K_{\theta }\left(x,y\right)$ before taking the squared modulus. The finite-source width therefore smooths the source-indexed response structure whenever the source profile is comparable to the fringe scale. Supplemental Section S3.2 gives the finite-source and finite-detector expressions used to connect the point model with finite experimental modes.

For a reciprocal linear optical medium, the Green function relating properly paired source and receiver modes obeys the usual reciprocity relation after the source and receiver are physically exchanged, with their mode normalizations treated consistently [30]. This physical statement is not the literal identity K(x,y)=K(y,x) in an unchanged asymmetric geometry. The statistical derivations below require only the non-negative transfer $|K_{\theta }{\left(x,y\right)|}^{2}$. Reciprocity is used when that transfer is interpreted as belonging to physically exchanged source–receiver experiments.

### 2.2. Programmed Source and Accepted Source–Detector Record

Let π(y) be the probability density used to program the source coordinate,

(6) ∫dyπ(y)=1,

and let η(x)≥0 denote detector acceptance or position-dependent efficiency. The unnormalized weight of a trial with source label *y* and accepted detector coordinate *x* is $\pi \left(y\right)\eta \left(x\right)|K_{\theta }{\left(x,y\right)|}^{2}$. Normalizing over both coordinates gives the joint density

(7) $$p_{\theta }\left(x,y\right)=\frac{\pi \left(y\right)\eta \left(x\right)|K_{\theta }{\left(x,y\right)|}^{2}}{Z_{\theta }},\;Z_{\theta }=\;\int \int \;dx\;dy\;\pi \left(y\right)\eta \left(x\right){\left|K_{\theta }\left(x,y\right)\right|}^{2}.$$

Thus, $Z_{\theta }$ is the total accepted optical weight for the chosen source program and detector acceptance. Equation (7) describes the distribution conditioned on an accepted detection event. If information is instead normalized per launched trial, the no-click outcome must be retained as an additional event; that normalization is different from the accepted-record analysis described here.

The detector and source marginals of the accepted data are

(8) $$p_{X,\theta }\left(x\right)=\int dy\;p_{\theta }\left(x,y\right),\;p_{Y,\theta }\left(y\right)=\int dx\;p_{\theta }\left(x,y\right).$$

The accepted-source marginal is generally not the programmed prior. Substitution of Equation (7) gives

(9) $$p_{Y,\theta }\left(y\right)=\frac{\pi \left(y\right)\int dx\;\eta \left(x\right)|K_{\theta }{\left(x,y\right)|}^{2}}{Z_{\theta }}.$$

Equation (9) identifies a physically important distinction between preparation and observation. The programmed prior π(y) describes how often source settings are launched, whereas $p_{Y,\theta }\left(y\right)$ describes how often those settings appear among accepted detections. A source label that couples more strongly to the accepted detector region is therefore over-represented in the accepted record. This acceptance-induced reshaping is the origin of the correction term that appears later in the averaged information gain.

### 2.3. Conditional Readouts in Young and TRY Measurements

A conventional Young measurement fixes the source at $y_{0}$ and records the detector coordinate. Conditioning Equation (7) on $Y=y_{0}$ gives

(10) $$\begin{array}{c} p_{\theta }\left(x|y_{0}\right)=\frac{p_{\theta }\left(x,y_{0}\right)}{p_{Y,\theta }\left(y_{0}\right)}=\frac{\eta \left(x\right)|K_{\theta }\left(x,y_{0}\right){|}^{2}}{\int dx^{\prime }\;\eta \left(x^{\prime }\right){\left|K_{\theta }\left(x^{\prime },y_{0}\right)\right|}^{2}}. \end{array}$$

The source prior cancels because the source setting is already fixed. Equation (10) is the canonical detector-plane Young probability law within the accepted detector region.

TRY performs the opposite conditioning. The detector channel is fixed at $x_{0}$, and the source label is retained. Conditioning Equation (7) on $X=x_{0}$ gives

(11) $$\begin{array}{c} p_{\theta }\left(y|x_{0}\right)=\frac{p_{\theta }\left(x_{0},y\right)}{p_{X,\theta }\left(x_{0}\right)}=\frac{\pi \left(y\right)|K_{\theta }\left(x_{0},y\right){|}^{2}}{\int dy^{\prime }\;\pi \left(y^{\prime }\right){\left|K_{\theta }\left(x_{0},y^{\prime }\right)\right|}^{2}}. \end{array}$$

The factor $\eta (x_{0})$ cancels because the same detector channel appears in the numerator and denominator. The notation $p_{\theta }\left(y|x_{0}\right)$ denotes a regular conditional density. Operationally, one first conditions on a finite detector region $D_{\epsilon }\left(x_{0}\right)$,

(12) $$p_{\theta ,\epsilon }\left(y|x_{0}\right)=\frac{\pi \left(y\right)\int_{D_{\epsilon }\left(x_{0}\right)}\;dx\;\eta \left(x\right){\left|K_{\theta }\left(x,y\right)\right|}^{2}}{\int dy^{\prime }\;\pi \left(y^{\prime }\right)\int_{D_{\epsilon }\left(x_{0}\right)}\;dx\;\eta \left(x\right){\left|K_{\theta }\left(x,y^{\prime }\right)\right|}^{2}},$$

and then takes the narrow-pixel limit ϵ→0 when the transfer is continuous and the denominator is nonzero. The explicit finite-pixel formulation is given in Supplemental Section S3.

Equations (10) and (11) are derived from the same joint distribution, but they normalize along different coordinates. A source-ensemble detector marginal, $p_{X,\theta }\left(x\right)$, is different from both conditionals. This distinction is the complete statistical basis for the entropy comparison below.

### 2.4. Discrete Experimental Form

Actual measurements use a finite collection of source settings and detector channels. Let $y_{j}$ (j=1,…,M) be the programmed source labels and $x_{i}$ (i=1,…,N) the detector channels. Let $\pi_{j}$ be the source probability, $\eta_{i}$ the detector acceptance, and $K_{ij}=K_{\theta }\left(x_{i},y_{j}\right)$ is the optical amplitude for that source–detector pair. The discrete joint probability is

(13) $$P_{ij}=\frac{\pi_{j}\eta_{i}{\left|K_{ij}\right|}^{2}}{Z},\;Z=\sum_{r=1}^{N}\sum_{s=1}^{M}\pi_{s}\eta_{r}{\left|K_{rs}\right|}^{2}.$$

The marginals are

(14) $$P_{i}^{X}=\sum_{j=1}^{M}P_{ij},\;P_{j}^{Y}=\sum_{i=1}^{N}P_{ij}.$$

The fixed-source and fixed-detector conditionals follow directly from the joint table:

(15) $$P_{i|j_{0}}=\frac{P_{ij_{0}}}{P_{j_{0}}^{Y}},\;P_{j|i_{0}}=\frac{P_{i_{0}j}}{P_{i_{0}}^{X}}.$$

For an experimental count matrix, Equations (13)–(15) correspond directly to the normalization of the accepted joint counts, summation over one index to obtain the marginals, and normalization of a selected column or row to obtain the two conditional readouts. The numerical entropies and information quantities below are computed from these probabilities.

Table 1 summarizes the probability laws used in the analysis and specifies the distinction between the detector marginal of a programmed ensemble and the canonical fixed-source Young distribution.

## 3. Measurement Entropy and Conditional Information

The joint table $P_{ij}$ now fixes the statistical object from which every information quantity is computed. This section connects standard information measures to the experimentally distinct reductions in Table 1. The order is important: entropy and information are assigned only after the probability law and the variables retained in the record have been specified.

### 3.1. Entropies of the Measured Reductions

We use natural logarithms, so information is measured in nats. The detector and source marginals have Shannon entropies

(16) $$H_{X}=-\sum_{i=1}^{N}P_{i}^{X}\ln P_{i}^{X},\;H_{Y}=-\sum_{j=1}^{M}P_{j}^{Y}\ln P_{j}^{Y}.$$

For a selected source $j_{0}$ and a selected detector $i_{0}$, the corresponding single-condition entropies are

(17) $$H_{X|j_{0}}=-\sum_{i=1}^{N}P_{i|j_{0}}\ln P_{i|j_{0}},\;H_{Y|i_{0}}=-\sum_{j=1}^{M}P_{j|i_{0}}\ln P_{j|i_{0}}.$$

These four quantities describe different probability distributions. In particular, $H_{X}$ is not the entropy of a canonical fixed-source Young experiment unless the source program contains only one source setting.

The ensemble conditional entropies average the selected conditional entropies with the corresponding marginal probabilities,

(18) $$\begin{array}{cc} H(X|Y) & =-\sum_{i,j}P_{ij}\ln P_{i|j}=\sum_{j}P_{j}^{Y}H\left(X|j\right),\\ H(Y|X) & =-\sum_{i,j}P_{ij}\ln P_{j|i}=\sum_{i}P_{i}^{X}H\left(Y|i\right). \end{array}$$

The joint entropy

(19) $$H\left(X,Y\right)=-\sum_{i,j}P_{ij}\ln P_{ij}$$

obeys the standard Shannon chain rule [19,20,22]. Expanding it in either conditioning order gives

(20) $$H\left(X,Y\right)=H_{Y}+H\left(X|Y\right)=H_{X}+H\left(Y|X\right).$$

Therefore,

(21) $$H\left(X|Y\right)-H\left(Y|X\right)=H_{X}-H_{Y}.$$

Equation (21) concerns the outcome-averaged conditional entropies. It does not impose an equality between $H_{X|j_{0}}$ and $H_{Y|i_{0}}$ for two selected experimental settings. Supplemental Section S4 derives Equations (20) and (21) directly from Equation (13).

The differential entropy for a continuous coordinate changes under a rescaling or a more general change in variables [28]. Operational measurement models therefore benefit from specifying the variables that are actually recorded [17]. The continuous densities in Section 2 define the optical probability law, whereas the discrete probabilities in Section 2.4 correspond to finite source settings and detector channels and are used for the Shannon entropies reported below. Mutual information and KL divergence retain their invariant meaning when continuous densities are transformed consistently [22].

### 3.2. Information Selected by One Fixed Detector

The TRY conditional $P_{j|i_{0}}$ is the distribution of source labels among events registered by detector channel $i_{0}$. The programmed distribution before propagation is $\pi_{j}$. Their Kullback–Leibler divergence therefore gives the prior-relative conditional information selected by that detector outcome:

(22) $$G_{\pi }\left(i_{0}\right)=D_{\mathrm{KL}}\;\left(P_{\cdot |i_{0}}∥\pi \right)=\sum_{j=1}^{M}P_{j|i_{0}}\ln\frac{P_{j|i_{0}}}{\pi_{j}}.$$

This is a standard information-theoretic quantity [21,22]. In the TRY measurement, it has a direct operational meaning: it quantifies how strongly the selected detector outcome changes the relative weights of the source labels that were programmed before propagation.

If all *M* source labels are programmed equally, $\pi_{j}=1/M$, Equation (22) becomes

(23) $$\begin{array}{c} G_{\pi }\left(i_{0}\right)=\sum_{j}P_{j|i_{0}}\ln\;\left(MP_{j|i_{0}}\right)=\ln M+\sum_{j}P_{j|i_{0}}\ln P_{j|i_{0}}=\ln M-H_{Y|i_{0}}. \end{array}$$

Thus, for an equally programmed finite-source basis, the entropy deficit $\ln M-H_{Y|i_{0}}$ is exactly the information gained about the prepared source label after selecting detector channel $i_{0}$. A value of zero means that the selected detector leaves all programmed source labels equally plausible; a larger value means that the detector outcome concentrates the accepted record onto a smaller subset of source settings. For a nonuniform source prior, Equation (22) remains the appropriate measure because the reference uncertainty is no longer simply lnM.

### 3.3. Mutual Information and Accepted-Source Correction

The mutual information of the complete accepted source–detector record is

(24) $$I\left(X;Y\right)=\sum_{i,j}P_{ij}\ln\frac{P_{ij}}{P_{i}^{X}P_{j}^{Y}}.$$

Using $P_{ij}=P_{i}^{X}P_{j|i}$ gives

(25) $$I\left(X;Y\right)=\sum_{i}P_{i}^{X}D_{\mathrm{KL}}\;\left(P_{\cdot |i}∥P^{Y}\right),$$

where $P^{Y}=\left\{P_{j}^{Y}\right\}$ is the source marginal in the accepted data. Equivalently,

(26) $$I\left(X;Y\right)=H_{Y}-H\left(Y|X\right)=H_{X}-H\left(X|Y\right).$$

The equality I(X;Y)=I(Y;X) follows from these standard definitions. It is not an additional optical reciprocity law.

Equation (25) also exposes an important reference-distribution difference. The local quantity $G_{\pi }\left(i\right)$ in Equation (22) compares the detector-conditioned source distribution with the *programmed prior* π. Mutual information compares it with the *accepted source marginal* $P^{Y}$. These distributions coincide only when the total accepted transfer is independent of source label.

To make this distinction quantitative, average Equation (22) over all accepted detector outcomes:

(27) $${\bar{G}}_{\pi }=\sum_{i}P_{i}^{X}D_{\mathrm{KL}}\;\left(P_{\cdot |i}∥\pi \right).$$

Insert $P_{j}^{Y}$ into the logarithm, and use $\sum_{i}P_{ij}=P_{j}^{Y}$. The resulting decomposition is

(28) $${\bar{G}}_{\pi }=I\left(X;Y\right)+D_{\mathrm{KL}}\;\left(P^{Y}∥\pi \right).$$

Equation (28) is the standard decomposition of the expected information gain used in Bayesian experimental design [40,41,42]. Its two terms have different experimental meanings here. The mutual information I(X;Y) measures the correlation between retained source labels and detector outcomes within the accepted record. The second term, $D_{\mathrm{KL}}\left(P^{Y}∥\pi \right)$, measures the selection bias introduced by the optical transfer and detector acceptance before any particular detector outcome is specified. This separation distinguishes information carried by source–detector correlation from information created by preferential acceptance of some source settings. A line-by-line derivation is given in Supplemental Section S4.

### 3.4. Finite-Count Estimation

The entropies above are the properties of the underlying probability distributions. In an experiment, they are estimated from finite photon counts. If $n_{i}$ counts are observed in $K_{+}$ occupied bins, with total count $n=\sum_{i}n_{i}$ and empirical frequencies ${\hat{p}}_{i}=n_{i}/n$, the plug-in estimator ${\hat{H}}_{\mathrm{ML}}=-\sum_{i}{\hat{p}}_{i}\ln{\hat{p}}_{i}$ is negatively biased at finite *n*. A standard first-order correction is the Miller–Madow estimator

(29) $${\hat{H}}_{\mathrm{MM}}={\hat{H}}_{\mathrm{ML}}+\frac{K_{+}-1}{2n},$$

for natural logarithms [43,44]. The mutual information and KL-based quantities obtained from empirical histograms also have finite-sample bias, particularly when the number of bins is not small compared with the number of detected events [45]. The numerical curves below therefore represent population probabilities generated by the optical model, not finite-count estimates. For experimental use, the binning and estimator must be reported together with the detected sample size; Supplemental Section S5.3 illustrates the grid dependence of the raw entropy and the convergence of the corresponding entropy deficit.

### 3.5. Optical Reciprocity and Statistical Conditioning

Optical reciprocity and statistical symmetry enter at different levels of the construction. Optical reciprocity constrains the Green function when properly paired source and receiver modes are physically exchanged [30,31,32]. The chain rule, Bayes’s rule, and I(X;Y)=I(Y;X) instead follow from probability theory once the joint law $P_{ij}$ is specified. Reciprocity therefore connects the source–receiver-exchanged optical geometries, whereas the source prior, detector acceptance, and chosen conditioning determine the measured probability distribution. Bayesian retrodiction remains prior-dependent [33,34,35].

## 4. Double-Slit Numerical Example

The numerical calculations use the double-slit response in Equation (5). Define

(30) $$\xi =\frac{y}{\lambda L_{s}/d},\;\zeta =\frac{x}{\lambda L_{d}/d}.$$

For notational economy, we henceforth write $\varphi \equiv \Delta \phi_{\theta }$. Equation (4) then becomes

(31) ΔΦ=−2π(ξ+ζ)+φ,

and the normalized transfer weight is

(32) $$1+V\cos\;\left[2\pi (\xi +\zeta )-\varphi \right],\;V=\frac{2a_{+}a_{-}}{a_{+}^{2}+a_{-}^{2}}.$$

The numerical calculations use this Fresnel-derived transfer directly.

**Figure 2:**
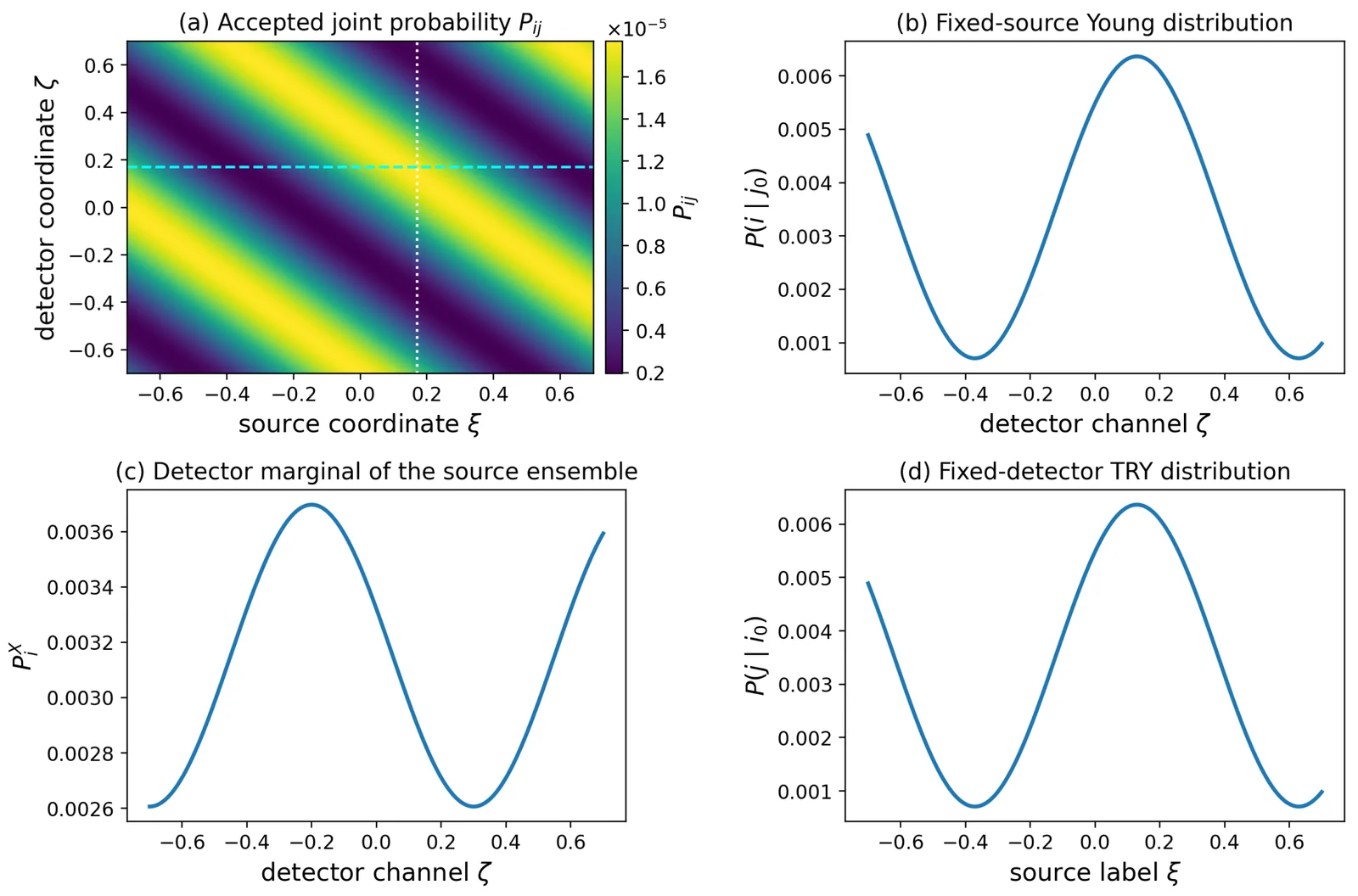
Probability reductions for matched-grid double-slit example. (**a**) Accepted joint probability $P_{ij}$; the dotted vertical line and dashed horizontal line mark the selected source and detector. (**b**) Fixed-source Young conditional $P(i|j_{0})$. (**c**) Detector marginal $P_{i}^{X}$. (**d**) Fixed-detector TRY conditional $P(j|i_{0})$. The calculation uses M=N=321 points on ξ,ζ∈[−0.70,0.70], uniform source programming and detector acceptance, φ=0.60π, V=0.80, and $\xi_{0}=\zeta_{0}≃0.17$. In this symmetric matched-grid case, the two conditionals in panels (**b**,**d**) are identical up to their axis labels.

The canonical calculation uses identical source and detector grids: M=N=321 points on [−0.70,0.70], with uniform source programming and detector acceptance. The selected source and detector are the grid points nearest $\xi_{0}=\zeta_{0}=0.17$. Figure 2 uses φ=0.60π and V=0.80. Because Equation (32) depends on ξ and ζ only through ξ+ζ, this symmetric discretization preserves the optical symmetry: the fixed-source Young and fixed-detector TRY conditionals have the same shape and the same entropy. The example therefore isolates detector conditioning without introducing numerical asymmetry through different windows or bin counts.

**Figure 3:**
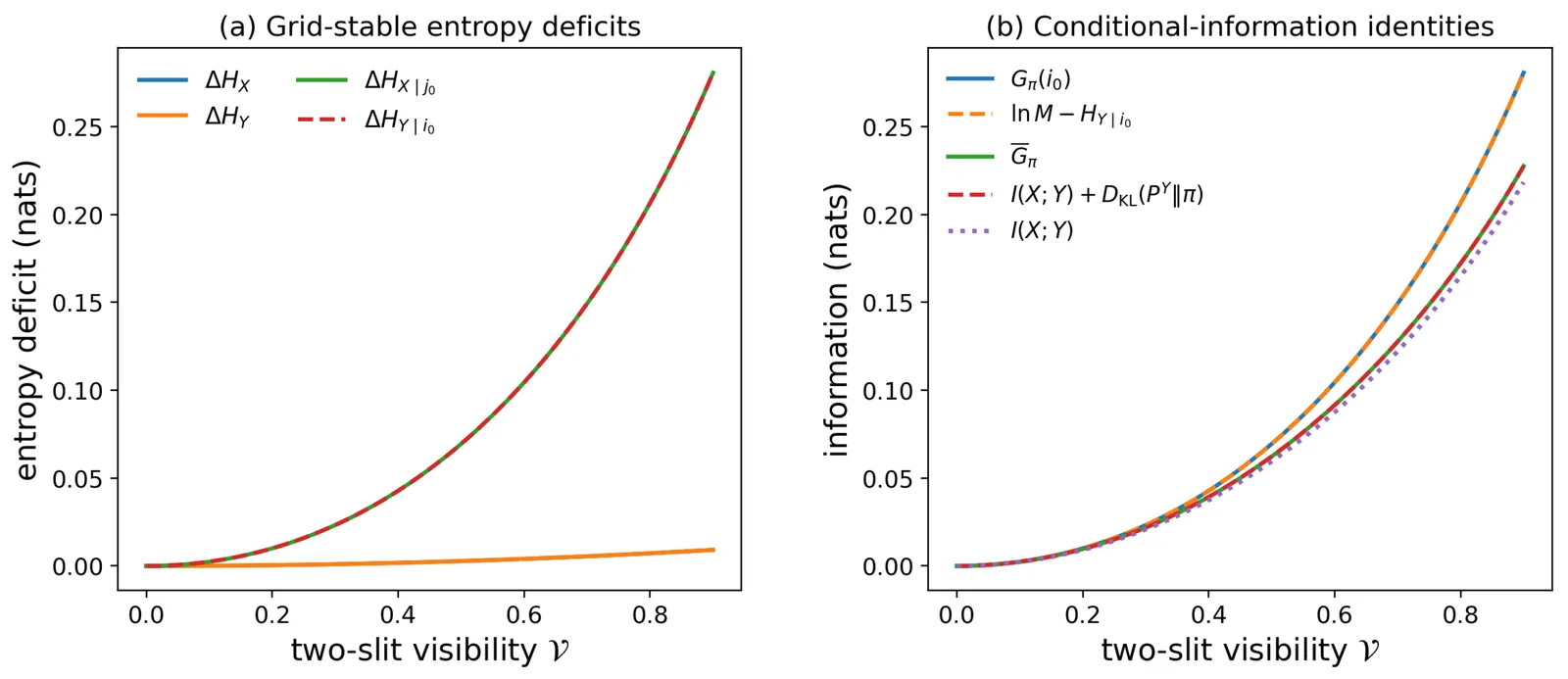
Grid-stable entropy deficits and conditional information versus the two-slit visibility V for the matched-grid example. (**a**) Entropy deficits relative to the corresponding uniform distribution. The fixed-source and fixed-detector conditional curves overlap in the symmetric geometry. (**b**) Prior-relative conditional information, its uniform-prior entropy form, the outcome-averaged information gain, and mutual information. The same grid, phase, selected source and detector, source prior, and detector acceptance as in Figure 2 are used, while 0≤V≤0.90. Supplemental Section S5 gives the numerical sums and a grid-refinement check.

Raw discrete entropies contain the familiar resolution term lnM or lnN and therefore grow as the grid is refined. Figure 3a instead plots four entropy deficits relative to uniform distributions on the same supports: $\Delta H_{X}\equiv \ln N-H_{X}$, $\Delta H_{Y}\equiv \ln M-H_{Y}$, $\Delta H_{X|j_{0}}\equiv \ln N-H_{X|j_{0}}$, and $\Delta H_{Y|i_{0}}\equiv \ln M-H_{Y|i_{0}}$. These deficits measure concentration relative to a uniform record and remain stable as a fixed physical window is sampled more finely. The overlapping conditional curves show that the symmetric kernel does not create an entropy difference between the matched Young and TRY conditionals. What changes with increasing visibility is the concentration of each distribution relative to its uniform reference.

Figure 3b connects that concentration directly to conditional information. For the uniform source prior, $G_{\pi }\left(i_{0}\right)=\Delta H_{Y|i_{0}}$, so the local KL gain and the source-space entropy deficit are the same quantity expressed in two languages. The outcome-averaged gain ${\bar{G}}_{\pi }$ is larger than the mutual information whenever detector acceptance reshapes the source ensemble, exactly by the correction $D_{\mathrm{KL}}\left(P^{Y}∥\pi \right)$. In the canonical symmetric configuration, the selected-detector gain reaches about 0.28 nats, approximately 4.9% of ln321. The modest size is physically informative: a uniform prior combined with the smooth finite-visibility double-slit response only weakly discriminates among source labels. Stronger source discrimination requires structured source programming, a more selective detector mode, or a different operating point, not a different information identity.

## 5. Local Sensitivity and Optical Coherence

The detector-conditioned entropy quantifies the spread of the source-label distribution at a fixed optical parameter. Two complementary properties of the same experiment require different descriptors: the local sensitivity of that distribution to a parameter perturbation and the coherence of the optical field before detection. Fisher information quantifies the first property, whereas coherence entropy quantifies the second. Evaluating all three quantities in the same double-slit model makes their distinct physical roles explicit.

For a fixed detector channel, the conditional law defines the standard Fisher information for a parameter θ. With a θ-independent source prior, the score is

(33) $$s_{\theta }\left(j|i_{0}\right)=\partial_{\theta }\ln P_{\theta }\left(j|i_{0}\right),$$

and the normalization of the conditional distribution yields the centered form

(34) $$s_{\theta }\left(j|i_{0}\right)=\partial_{\theta }\ln{\left|K_{\theta }\left(x_{i_{0}},y_{j}\right)\right|}^{2}-{\langle \partial_{\theta }\ln{\left|K_{\theta }\left(x_{i_{0}},y\right)\right|}^{2}\rangle }_{Y|i_{0}}.$$

**Figure 4:**
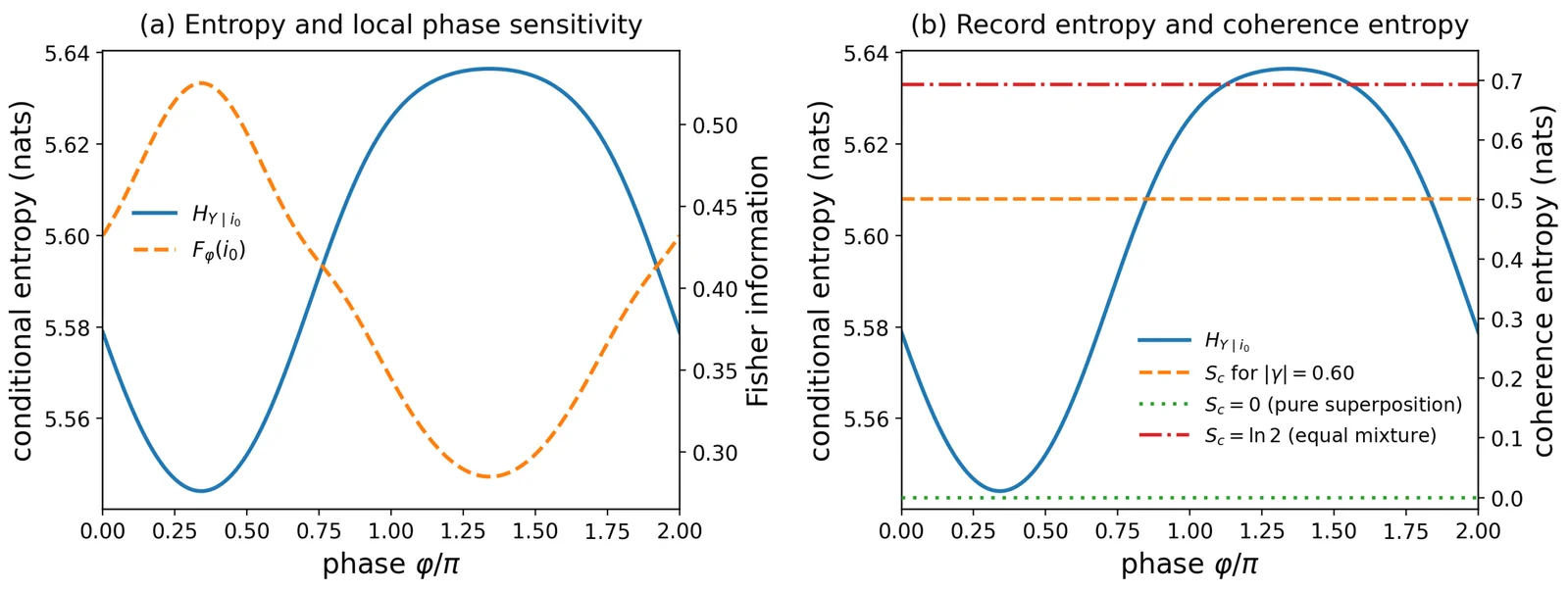
Complementary information measures for the same fixed-grid double-slit phase scan at V=0.80. (**a**) Conditional source-record entropy $H_{Y|i_{0}}$ and phase Fisher information $F_{\varphi }\left(i_{0}\right)$. Entropy measures the spread of the conditional distribution, whereas Fisher information measures its local sensitivity to φ; their different phase dependences show that these properties are not interchangeable. (**b**) The same record entropy compared with the two-mode coherence entropy. For |γ|=0.60, $S_{c}=0.500$ nats is invariant under the phase-only unitary evolution, while $H_{Y|i_{0}}$ changes with detector-conditioned source probabilities. The pure-superposition limit $S_{c}=0$ and incoherent equal-mixture limit $S_{c}=\ln2$ are shown for reference.

The Fisher information per accepted event is therefore

(35) $$F_{\theta }\left(i_{0}\right)=\sum_{j}P_{\theta }\left(j|i_{0}\right)s_{\theta }^{2}\left(j|i_{0}\right),$$

a standard estimation theory result [23,24,25]; the related connections between Fisher information and diffraction optics have also been discussed [46]. Figure 4a evaluates $H_{Y|i_{0}}$ and the phase Fisher information $F_{\varphi }\left(i_{0}\right)$ on the same fixed grid while φ is scanned. The grid is unchanged, so the additive resolution term in the discrete entropy is constant. The two curves respond differently because they probe different geometric features of the likelihood: entropy depends on the instantaneous spread of probability mass over source labels, whereas Fisher information depends on the squared local slope of that probability mass with respect to phase. Their opposite motion over part of the cycle therefore does not signal a universal inverse relation; it demonstrates that a concentrated distribution need not be locally sensitive and a locally sensitive distribution need not have minimal entropy. The calculation uses V=0.80, for which all sampled probabilities remain positive. If an exact destructive-response zero enters the support, the logarithmic score becomes singular, and the equivalent form $\sum_{j}{\left(\partial_{\theta }P_{j}\right)}^{2}/P_{j}$ must be interpreted by a limit on the probability support. Differential source-basis estimation and null-constrained designs provide complementary analyses of these high-sensitivity regimes [11,47].

The source labels used above are classical preparation settings, not coherent basis states. A coherent superposition and an incoherent mixture therefore require a separate field-state description. For a two-mode coherence operator

(36) $$\rho_{c}=\frac{1}{2}\left(\begin{array}{cc} 1 & \gamma \\ \gamma^{*} & 1 \end{array}\right),\;\mu_{\pm }=\frac{1\pm |\gamma |}{2},$$

the coherence entropy is

(37) $$S_{c}=-\sum_{\pm }\mu_{\pm }\ln\mu_{\pm }.$$

A pure equal-amplitude superposition has |γ|=1 and $S_{c}=0$, whereas an incoherent equal mixture has γ=0 and $S_{c}=\ln2$. Here, |γ| is the magnitude of the normalized mutual coherence between the two modes, and $\mu_{\pm }=\left(1\pm |\gamma |\right)/2$ are the eigenvalues of $\rho_{c}$. Under ideal unitary propagation, $\rho_{c}\mapsto U\rho_{c}U^{†}$, so these eigenvalues and $S_{c}$ are unchanged. Figure 4b makes the separation quantitative. For |γ|=0.60, $S_{c}=0.500$ nats is phase-independent, while $H_{Y|i_{0}}$ varies because conditioning redistributes probability among classical source labels. The pure-superposition and equal-mixture limits bound the coherence entropy but do not bound the record entropy. Optical filtering, loss, or postselection can change the normalized coherence spectrum and therefore $S_{c}$ [48,49,50,51]; a phase-only unitary cannot. Thus, record entropy and coherence entropy answer complementary, not interchangeable, questions.

## 6. Discussion

The accepted joint distribution in Equation (7) is the organizing object of the theory. Once the source prior, optical transfer, and detector acceptance are specified, the detector marginal, accepted source marginal, fixed-source Young conditional, and fixed-detector TRY conditional are obtained by ordinary reduction of the same joint record. Standard information measures then acquire unambiguous experimental meanings: Shannon entropy describes uncertainty within a chosen reduction, KL divergence measures how a detector outcome reweights the programmed source labels, mutual information measures the source–detector correlation in the accepted record, and Fisher information measures the local parameter sensitivity of a selected conditional likelihood.

The symmetric matched-grid example shows both the strength and the limitation of a direct entropy comparison. Because the ideal double-slit transfer depends only on ξ+ζ, matched Young and TRY conditionals coincide; there is no intrinsic entropy asymmetry to extract from that special kernel. The useful quantity is instead the amount of concentration produced by conditioning relative to a specified source prior. Grid-stable entropy deficits and KL divergences capture this concentration without confusing it with bin resolution. The modest gain in the canonical case also identifies where design freedom enters: the programmed prior, detector acceptance, and operating point can be chosen to emphasize the source labels that couple differently to the fixed detector.

The joint-record formulation also places the entropy analysis within the broader development of TRY. The original two-slit work established the fixed-detector/source-scanned geometry [8]; the statistical foundation study formalized the source-labeled count record and fixed-detector likelihood [9]; Bayesian source-prior engineering treats preparation design [10]; differential source-basis encoding applies the same likelihood structure to local estimation [11]. Together, these results show how the same optical architecture supports complementary descriptions at the levels of propagation, statistics, information, and estimation.

The classical source–detector record and a post-measurement quantum state are different objects. Photodetection may physically absorb an excitation, whereas Equations (7)–(15) describe only the statistics of accepted source and detector labels and contain no quantum state-update map or sequence of quantum instruments. Questions of invasiveness, Leggett–Garg/Wigner realism, and measurement-induced disturbance therefore belong to an extended quantum description [36,37,38]. Within the present optical model, the two-mode coherence calculation instead isolates the relation between field coherence and uncertainty in the classical source-label record.

## 7. Conclusions

We described measurement entropy and conditional information in TRY through one accepted source–detector probability law. Fresnel propagation supplies the optical transfer, source programming supplies the prior, and detector acceptance specifies which events enter the record. Young and TRY readouts then appear as different conditionals of that common distribution, with the source and detector marginals obtained by summation or integration.

For the symmetric matched-grid double-slit example, the fixed-source Young and fixed-detector TRY conditionals have identical entropies. The physically meaningful distinction is therefore not a universal entropy imbalance but the different preparation and conditioning operations that define the measured records. Entropy deficits and KL information gains quantify how strongly a fixed detector concentrates the programmed source distribution, while the decomposition of the averaged gain separates source–detector correlation from the acceptance-induced reshaping of the source ensemble.

Finite slit and source widths, detector pixels, and finite photon counts establish the route from the ideal model to experiment. The phase scan further shows that record entropy, Fisher information, and optical coherence entropy describe complementary properties: distributional spread, local parameter sensitivity, and modal coherence. Keeping these meanings separate yields a compact and experimentally interpretable information-theoretic description of fixed-detector, source-programmed Young interferometry.

## Figures and Tables

**Table 1:** Probability distributions used in this manuscript and how each is computed from the accepted joint table $P_{ij}$.

Statistical Object	Computation	Experimental Meaning
$P_{ij}$	Normalized complete accepted source–detector count table	Joint record retaining both programmed source label *j* and detector channel *i*
$P_{i}^{X}$	$\sum_{j}P_{ij}$	Detector marginal after source labels are discarded
$P_{j}^{Y}$	$\sum_{i}P_{ij}$	Source-label distribution within the accepted record; it can differ from $\pi_{j}$
$P_{i|j_{0}}$	$P_{ij_{0}}/P_{j_{0}}^{Y}$	Canonical Young distribution for one fixed source setting
$P_{j|i_{0}}$	$P_{i_{0}j}/P_{i_{0}}^{X}$	TRY source-label distribution selected by one fixed detector channel

## Data Availability

No experimental data sets were generated in this theoretical study. The equations, numerical parameters, and discrete probability sums required to reproduce the numerical results are provided in this article and Supplementary Materials.

## Supplementary Materials

The following supporting information can be downloaded at: https://www.mdpi.com/article/10.3390/e28101093/s1, Supplementary File S1: Detailed derivations; finite-slit, finite-source, and finite-pixel generalizations; entropy and information identities; grid-refinement and finite-count notes; Fisher information derivation; coherence entropy calculation; and numerical evaluation.
