# Supplementary material for: Measurement Entropy and Conditional Information in Time-Reversed Young Interferometry
Source: Entropy (Basel). 2026 Oct 8;28(10):1093. doi: 10.3390/e28101093 (PMC13703189; doi:10.3390/e28101093)
Supplement: Supplementary file 1 [file entropy-28-01093-s001.zip › entropy-4576900-supplementary.pdf]

Supplemental Material for "Measurement Entropy and Conditional Information in Time-Reversed Young Interferometry"

Jianming Wen
Department of Electrical and Computer Engineering, Binghamton University, Binghamton, New York 13902, USA

S1. Notation and assumptions

This Supplemental Material collects the detailed derivations and numerical specifications used in the main text. The notation table is included only for quick reference. The optical wavelength is denoted by λ, while μ_n denotes an eigenvalue of the normalized coherence operator.

Symbol	Physical meaning	How it is obtained or used
x, y, u	Detector, source, and aperture transverse coordinates	Experimental coordinates in the three optical planes
L_d, L_s	Aperture-to-detector and source-to-aperture distances	Geometric distances of the Fresnel system
λ, k	Optical wavelength and wavenumber, $k = 2\pi/\lambda$	Illumination parameter
$T_\theta(u)$	Complex aperture transmission	Specified optical aperture, including any parameter dependence
$K_\theta(x, y)$	Source-to-detector scalar amplitude	Fresnel integral in Eq. (S1)
$\pi(y), \pi_j$	Programmed source probability density or discrete probability	Set by source dwell time or trial frequency
$\eta(x), \eta_i$	Detector acceptance or efficiency	Set by the detector window, pixel, or channel efficiency
Z_θ, Z	Total accepted normalization	Integral or sum of programmed, accepted optical weights
$p_\theta(x, y), P_{ij}$	Continuous or discrete joint source–detector probability	Normalized programmed and accepted optical weight
P_i^X, P_j^Y	Accepted detector and source marginals	Row or column sums of P_{ij}
$P_{i\|j}, P_{j\|i}$	Detector-given-source and source-given-detector conditionals	Normalized column or row of P_{ij}
H_X, H_Y	Marginal Shannon entropies	Direct sums over P_i^X or P_j^Y
$H_{X\|j_0}, H_{Y\|i_0}$	Entropies for one selected source or detector	Direct sums over $P_{i\|j_0}$ or $P_{j\|i_0}$
$I(X;Y)$	Mutual information of the complete accepted record	Eq. (S27)
$G_\pi(i)$	Conditional information relative to the programmed source prior	$D_{\mathrm{KL}}(P_{\cdot\|i} \\| \pi)$
Γ, ρ_c, μ_n	Cross-spectral density, normalized coherence operator, and its eigenvalues	Used only for coherence entropy, not measurement-record entropy

The source label is treated as a classical preparation variable. The optical model is scalar, linear, monochromatic, and paraxial. Reciprocity is invoked when the Green function is interpreted under physical source–receiver exchange. Once a normalized joint distribution has been defined, the probability and information identities below apply to any nonnegative source–detector transfer.

S2. From Fresnel propagation to the two-slit transfer

S2.1 General scalar kernel

For a source point y, aperture coordinate u, detector coordinate x, wavelength λ, and $k = 2\pi/\lambda$, Fresnel propagation gives, apart from a coordinate-independent prefactor,

$$K_\theta(x, y) = \int \mathrm{d}u\, T_\theta(u) \exp\left[\frac{ik}{2L_s}(u - y)^2 + \frac{ik}{2L_d}(x - u)^2\right]. \tag{S1}$$

The omitted prefactor multiplies all source–detector amplitudes by the same constant and therefore cancels from every normalized probability in the main text.

S2.2 Narrow double slit

For two narrow transmitting elements at $u_+ = d/2$ and $u_- = -d/2$,

$$T_\theta(u) = a_+ e^{i\phi_+(\theta)}\delta(u - d/2) + a_- e^{i\phi_-(\theta)}\delta(u + d/2). \tag{S2}$$

Substitution into Eq. (S1) gives

$$\begin{aligned} K_\theta(x, y) &= a_+ e^{i\phi_+} \int \mathrm{d}u\, \delta(u - d/2) e^{i\Phi(u;x,y)} + a_- e^{i\phi_-} \int \mathrm{d}u\, \delta(u + d/2) e^{i\Phi(u;x,y)} \\ &= C\left(a_+ e^{i\Phi_+(x,y)} + a_- e^{i\Phi_-(x,y)}\right), \end{aligned} \tag{S3}$$

where

$$\Phi_\pm(x, y) = \frac{k}{2L_s}(\pm d/2 - y)^2 + \frac{k}{2L_d}(x \mp d/2)^2 + \phi_\pm(\theta). \tag{S4}$$

The two-path phase difference is

$$\begin{aligned} \Delta\Phi_\theta(x, y) &= \Phi_+(x, y) - \Phi_-(x, y) \\ &= \frac{k}{2L_s}\left[(d/2 - y)^2 - (-d/2 - y)^2\right] + \frac{k}{2L_d}\left[(x - d/2)^2 - (x + d/2)^2\right] + \Delta\phi_\theta. \end{aligned} \tag{S5}$$

The two quadratic differences are

$$(d/2 - y)^2 - (-d/2 - y)^2 = -2dy, \qquad (x - d/2)^2 - (x + d/2)^2 = -2dx. \tag{S6}$$

Therefore

$$\Delta\Phi_\theta(x, y) = -kd\left(\frac{y}{L_s} + \frac{x}{L_d}\right) + \Delta\phi_\theta. \tag{S7}$$

Taking the squared magnitude of Eq. (S3) gives

$$|K_\theta(x, y)|^2 = |C|^2\left[a_+^2 + a_-^2 + 2a_+a_- \cos\Delta\Phi_\theta(x, y)\right]. \tag{S8}$$

This transfer generates the numerical figures in the main text.

S2.3 Dimensionless fringe coordinates used in the figures

Define the source and detector fringe scales

$$\Delta y = \frac{\lambda L_s}{d}, \qquad \Delta x = \frac{\lambda L_d}{d}, \tag{S9}$$

and the dimensionless coordinates

$$\xi = \frac{y}{\Delta y}, \qquad \zeta = \frac{x}{\Delta x}. \tag{S10}$$

Since $kdy/L_s = 2\pi\xi$ and $kdx/L_d = 2\pi\zeta$, Eq. (S7) becomes

$$\Delta\Phi = -2\pi(\xi + \zeta) + \varphi, \qquad \varphi \equiv \Delta\phi_\theta. \tag{S11}$$

Dividing Eq. (S8) by $|C|^2(a_+^2 + a_-^2)$ gives the dimensionless transfer weight

$$w(\zeta, \xi) = 1 + \mathcal{V}\cos[2\pi(\xi + \zeta) - \varphi], \qquad \mathcal{V} = \frac{2a_+a_-}{a_+^2 + a_-^2}. \tag{S12}$$

No additional optical model is used in the numerical calculation.

S2.4 Finite slit width

The delta-function slit model is the narrow-aperture limit of a finite transmission window. For two equal rectangular slits of width w,

$$T_\theta(u) = a_+e^{i\phi_+}\,\Pi\!\left(\frac{u - d/2}{w}\right) + a_-e^{i\phi_-}\,\Pi\!\left(\frac{u + d/2}{w}\right), \tag{S13}$$

where $\Pi(v) = 1$ for $|v| \le 1/2$ and zero otherwise. Substitution into the Fresnel kernel gives a sum of two finite Fresnel integrals. In the Fraunhofer limit the common single-slit factor is

$$\operatorname{sinc}\left[\frac{kw}{2}\left(\frac{y}{L_s} + \frac{x}{L_d}\right)\right], \tag{S14}$$

so that the intensity acquires the corresponding sinc-squared envelope multiplying the two-path interference term. Thus finite slit width smooths both the detector-space and source-space conditionals. The delta-slit expressions of the main text are recovered as $w \to 0$ or whenever the envelope varies negligibly over the sampled window.

S3. From optical transfer to conditional probabilities

S3.1 Continuous accepted distribution

Let $\pi(y)$ be the normalized source program and $\eta(x)$ the detector acceptance. Define

$$Z_\theta = \iint \mathrm{d}x\,\mathrm{d}y\,\pi(y)\eta(x)|K_\theta(x, y)|^2. \tag{S15}$$

Then

$$p_\theta(x, y) = \frac{\pi(y)\eta(x)|K_\theta(x, y)|^2}{Z_\theta} \tag{S16}$$

satisfies

$$\iint \mathrm{d}x\,\mathrm{d}y\,p_\theta(x,y) = 1. \tag{S17}$$

The marginals are

$$p_{X,\theta}(x) = \int \mathrm{d}y\,p_\theta(x,y), \qquad p_{Y,\theta}(y) = \int \mathrm{d}x\,p_\theta(x,y). \tag{S18}$$

For a source fixed at y_0,

$$\begin{aligned} p_\theta(x|y_0) &= \frac{p_\theta(x,y_0)}{p_{Y,\theta}(y_0)} \\ &= \frac{\pi(y_0)\eta(x)|K_\theta(x,y_0)|^2/Z_\theta}{\pi(y_0)\int \mathrm{d}x'\,\eta(x')|K_\theta(x',y_0)|^2/Z_\theta} \\ &= \frac{\eta(x)|K_\theta(x,y_0)|^2}{\int \mathrm{d}x'\,\eta(x')|K_\theta(x',y_0)|^2}. \end{aligned} \tag{S19}$$

The source-program factor and the global normalization cancel.

For a detector channel fixed at x_0,

$$\begin{aligned} p_\theta(y|x_0) &= \frac{p_\theta(x_0,y)}{p_{X,\theta}(x_0)} \\ &= \frac{\pi(y)\eta(x_0)|K_\theta(x_0,y)|^2/Z_\theta}{\eta(x_0)\int \mathrm{d}y'\,\pi(y')|K_\theta(x_0,y')|^2/Z_\theta} \\ &= \frac{\pi(y)|K_\theta(x_0,y)|^2}{\int \mathrm{d}y'\,\pi(y')|K_\theta(x_0,y')|^2}. \end{aligned} \tag{S20}$$

The detector acceptance at the selected channel and the global normalization cancel. Equations (S19) and (S20) are the two conditional normalizations used throughout the main text.

S3.2 Finite detector pixel or mode

If detector channel i covers a finite region D_i with local efficiency $\eta_i(x)$, its accepted transfer from source setting y_j is obtained by the integral

$$A_{ij} = \int_{D_i} \mathrm{d}x\,\eta_i(x)|K_\theta(x,y_j)|^2. \tag{S21}$$

For discrete source probabilities π_j, the finite-pixel joint probability is

$$P_{ij} = \frac{\pi_j A_{ij}}{\sum_{r,s}\pi_s A_{rs}}. \tag{S22}$$

The point-channel expression in the main text is the narrow-pixel limit $A_{ij} \propto \eta_i|K_\theta(x_i,y_j)|^2$. All subsequent probability identities are unchanged because they depend only on the normalized matrix P_{ij}.

S3.3 Discrete marginal and conditional probabilities

From a normalized joint table P_{ij},

$$P_i^X = \sum_j P_{ij}, \qquad P_j^Y = \sum_i P_{ij}. \tag{S23}$$

The fixed-source Young distribution is

$$P_{i|j_0} = \frac{P_{ij_0}}{P^Y_{j_0}}, \qquad \sum_i P_{i|j_0} = 1, \tag{S24}$$

and the fixed-detector TRY distribution is

$$P_{j|i_0} = \frac{P_{i_0 j}}{P^X_{i_0}}, \qquad \sum_j P_{j|i_0} = 1. \tag{S25}$$

For an experimental count matrix, Eqs. (S23)–(S25) correspond to marginalization of the normalized joint counts and normalization of the selected source or detector slice.

S4. Entropy and information identities

S4.1 Chain rule

The joint entropy is

$$H(X,Y) = -\sum_{i,j} P_{ij} \ln P_{ij}. \tag{S26}$$

Using $P_{ij} = P^Y_j P_{i|j}$,

$$\begin{aligned} H(X,Y) &= -\sum_{i,j} P_{ij} \ln(P^Y_j P_{i|j}) \\ &= -\sum_{i,j} P_{ij} \ln P^Y_j - \sum_{i,j} P_{ij} \ln P_{i|j} \\ &= -\sum_j P^Y_j \ln P^Y_j + H(X|Y) \\ &= H_Y + H(X|Y). \end{aligned} \tag{S27}$$

Using instead $P_{ij} = P^X_i P_{j|i}$ gives

$$H(X,Y) = H_X + H(Y|X). \tag{S28}$$

Equating Eqs. (S27) and (S28) gives the entropy balance in the main text.

S4.2 Mutual information

Start from

$$I(X;Y) = \sum_{i,j} P_{ij} \ln \frac{P_{ij}}{P^X_i P^Y_j}. \tag{S29}$$

Using $P_{ij} = P^X_i P_{j|i}$,

$$\begin{aligned} I(X;Y) &= \sum_{i,j} P_{ij} \ln \frac{P_{j|i}}{P^Y_j} \\ &= \sum_i P^X_i \sum_j P_{j|i} \ln \frac{P_{j|i}}{P^Y_j} \\ &= \sum_i P^X_i D_{\mathrm{KL}}(P_{\cdot|i} \| P^Y). \end{aligned} \tag{S30}$$

Expanding Eq. (S29) into the three logarithms $\ln P_{ij} - \ln P_i^X - \ln P_j^Y$ gives

$$I(X;Y) = H_X + H_Y - H(X,Y) = H_Y - H(Y|X) = H_X - H(X|Y). \tag{S31}$$

The symmetry under $X \leftrightarrow Y$ is therefore a property of the joint probability distribution.

S4.3 Fixed-detector prior-relative information

For a selected detector channel i_0,

$$G_\pi(i_0) = \sum_j P_{j|i_0} \ln \frac{P_{j|i_0}}{\pi_j}. \tag{S32}$$

For $\pi_j = 1/M$,

$$\begin{aligned} G_\pi(i_0) &= \sum_j P_{j|i_0} \ln P_{j|i_0} + \ln M \sum_j P_{j|i_0} \\ &= -H_{Y|i_0} + \ln M. \end{aligned} \tag{S33}$$

For a nonuniform prior, the corresponding entropy form is

$$G_\pi(i_0) = -H_{Y|i_0} - \sum_j P_{j|i_0} \ln \pi_j. \tag{S34}$$

Equation (S34) shows why the simple maximum-entropy subtraction is special to an equal source program.

S4.4 Outcome-averaged prior-relative information

Define

$$\overline{G}_\pi = \sum_i P_i^X D_{\mathrm{KL}}(P_{\cdot|i} \| \pi). \tag{S35}$$

Insert P_j^Y inside the logarithm:

$$\ln \frac{P_{j|i}}{\pi_j} = \ln \frac{P_{j|i}}{P_j^Y} + \ln \frac{P_j^Y}{\pi_j}. \tag{S36}$$

Then

$$\begin{aligned} \overline{G}_\pi &= \sum_{i,j} P_i^X P_{j|i} \ln \frac{P_{j|i}}{P_j^Y} + \sum_{i,j} P_i^X P_{j|i} \ln \frac{P_j^Y}{\pi_j} \\ &= I(X;Y) + \sum_j \left(\sum_i P_{ij} \right) \ln \frac{P_j^Y}{\pi_j} \\ &= I(X;Y) + D_{\mathrm{KL}}(P^Y \| \pi). \end{aligned} \tag{S37}$$

The second term vanishes if and only if the accepted source marginal equals the programmed prior on their common support.

S5. Numerical evaluation, grid refinement, and finite counts

S5.1 Matched-grid calculation

All numerical points in Figures 2 and 3 are generated from the dimensionless transfer in Eq. (S12). The source and detector coordinates use identical grids,

$$\xi, \zeta \in [-0.70, 0.70], \qquad M = N = 321, \tag{S38}$$

with uniform source probabilities and detector acceptance. The phase is $\varphi = 0.60\pi$, and the selected source and detector are the grid points nearest $\xi_0 = \zeta_0 = 0.17$. The transfer matrix is

$$w_{ij} = 1 + \mathcal{V} \cos[2\pi(\xi_j + \zeta_i) - \varphi]\,, \qquad P_{ij} = \frac{w_{ij}}{\sum_{r,s} w_{rs}}. \tag{S39}$$

Figure 2 uses $\mathcal{V} = 0.80$. Figure 3 uses 91 equally spaced values from $\mathcal{V} = 0$ to 0.90.

The raw discrete entropies grow logarithmically with the number of bins. The plotted quantities are therefore the entropy deficits

$$\Delta H_X = \ln N - H_X, \quad \Delta H_Y = \ln M - H_Y, \quad \Delta H_{X|j_0} = \ln N - H_{X|j_0}, \quad \Delta H_{Y|i_0} = \ln M - H_{Y|i_0}. \tag{S40}$$

For identical windows and spacings, the fixed-source and fixed-detector conditionals are identical functions of their respective coordinates, and therefore $\Delta H_{X|j_0} = \Delta H_{Y|i_0}$ when $\xi_0 = \zeta_0$.

S5.2 Grid refinement

Figure S1 shows the refinement behavior for the fixed-detector conditional at $\mathcal{V} = 0.80$ and $\varphi = 0.60\pi$. The source window remains $[-0.70, 0.70]$, the detector coordinate is fixed at $\zeta_{i_0} = 0.170625$, and $M = 41, 81, 161, 321, 641, 1281$. As the number of bins increases, the raw entropy $H_{Y|i_0}$ grows approximately as $\ln M$, whereas $\ln M - H_{Y|i_0}$ converges. This is the discrete counterpart of the coordinate-scale dependence of differential entropy discussed in the main text.

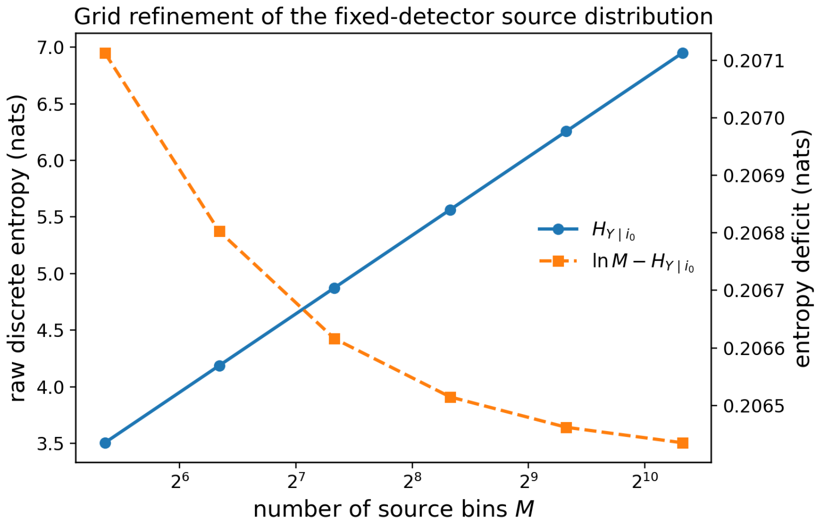

Figure S1: Grid-refinement check for the fixed-detector source-label distribution. The raw discrete entropy grows with the number of bins, whereas the entropy deficit relative to the uniform distribution converges.

S5.3 Finite-count estimation

If n_j counts are observed in the M source bins after fixing detector channel i_0, the empirical probabilities are $\hat{p}_j = n_j/n$, with $n = \sum_j n_j$. The plug-in entropy is

$$\hat{H}_{\mathrm{ML}} = -\sum_{j:n_j>0} \hat{p}_j \ln \hat{p}_j. \tag{S41}$$

For K_+ occupied bins, the first-order Miller–Madow correction is

$$\hat{H}_{\mathrm{MM}} = \hat{H}_{\mathrm{ML}} + \frac{K_+ - 1}{2n}. \tag{S42}$$

This correction is useful only when the sample is sufficiently large relative to the effective support; more general entropy and mutual-information estimators may be needed in sparse-count regimes. The main numerical figures display population quantities, so no estimator bias is present in those curves.

S6. Fisher information for the phase scan

For the phase parameter φ, the fixed-detector conditional used in Figure 4 is

$$P_\varphi(j|i_0) = \frac{1 + \mathcal{V}\cos[2\pi(\xi_j + \zeta_{i_0}) - \varphi]}{\sum_m \{1 + \mathcal{V}\cos[2\pi(\xi_m + \zeta_{i_0}) - \varphi]\}}. \tag{S43}$$

The score is $s_\varphi(j|i_0) = \partial_\varphi \ln P_\varphi(j|i_0)$ and the Fisher information is

$$F_\varphi(i_0) = \sum_j P_\varphi(j|i_0) s_\varphi^2(j|i_0) = \sum_j \frac{[\partial_\varphi P_\varphi(j|i_0)]^2}{P_\varphi(j|i_0)}. \tag{S44}$$

Figure 4 uses $\mathcal{V} = 0.80$ and 801 equally spaced phase values over $0 \le \varphi \le 2\pi$, so every probability is strictly positive and both forms are regular. At $\mathcal{V} = 1$, destructive-interference zeros can enter the sampled support. The logarithmic score then becomes singular at the zero, and the support-aware expression in Eq. (S44) must be interpreted by a limiting procedure. That singular regime is not used in the present entropy figures.

S7. Two-mode coherence entropy

For the normalized two-mode coherence operator

$$\rho_c = \frac{1}{2}\begin{pmatrix} 1 & \gamma \\ \gamma^* & 1 \end{pmatrix}, \tag{S45}$$

the eigenvalues are

$$\mu_\pm = \frac{1 \pm |\gamma|}{2}, \tag{S46}$$

and

$$S_c = -\mu_+ \ln \mu_+ - \mu_- \ln \mu_-. \tag{S47}$$

For $|\gamma| = 1$, the state is a pure equal-amplitude superposition and $S_c = 0$. For $\gamma = 0$, it is an incoherent equal mixture and $S_c = \ln 2$. The intermediate value used in Figure 4 is $|\gamma| = 0.60$, which gives $S_c = 0.5004$ nats. A unitary propagation $\rho_c' = U\rho_c U^\dagger$ leaves the eigenvalues unchanged, whereas filtering or loss followed by renormalization can change them. This coherence entropy is separate from the Shannon entropy of the classical source-label record.
